# Collective Dynamics of Belief Evolution under Cognitive Coherence and Social Conformity

**DOI:** 10.1371/journal.pone.0165910

**Published:** 2016-11-03

**Authors:** Nathaniel Rodriguez, Johan Bollen, Yong-Yeol Ahn

**Affiliations:** The Center for Complex Networks and Systems Research, School of Informatics and Computing, Indiana University, Bloomington, Indiana, United States of America; Universite de Namur, BELGIUM

## Abstract

Human history has been marked by social instability and conflict, often driven by the irreconcilability of opposing sets of beliefs, ideologies, and religious dogmas. The dynamics of belief systems has been studied mainly from two distinct perspectives, namely how cognitive biases lead to individual belief rigidity and how social influence leads to social conformity. Here we propose a unifying framework that connects cognitive and social forces together in order to study the dynamics of societal belief evolution. Each individual is endowed with a network of interacting beliefs that evolves through interaction with other individuals in a social network. The adoption of beliefs is affected by both internal coherence and social conformity. Our framework may offer explanations for how social transitions can arise in otherwise homogeneous populations, how small numbers of zealots with highly coherent beliefs can overturn societal consensus, and how belief rigidity protects fringe groups and cults against invasion from mainstream beliefs, allowing them to persist and even thrive in larger societies. Our results suggest that strong consensus may be insufficient to guarantee social stability, that the cognitive coherence of belief-systems is vital in determining their ability to spread, and that coherent belief-systems may pose a serious problem for resolving social polarization, due to their ability to prevent consensus even under high levels of social exposure. We argue that the inclusion of cognitive factors into a social model could provide a more complete picture of collective human dynamics.

## Introduction

Ideological conflict has been a major challenge for human societies [1]. For instance, when post World-War I Germany was marked by economic depression and social trauma, ideological fringe groups like the National-Socialist Party and the Communist Party of Germany made their way into mainstream politics to eventually dominate the political landscape [2–4]. This process of ideological upheaval eventually led to World War II, one of the deadliest conflicts in human history. Similarly, 14th and 15th century Europe was torn by sharp religious transitions and accompanying widespread conflicts, such as the Thirty Years’ War [5]. The abundance of such ideological transitions in history raises questions: are they driven by common psycho-social mechanisms? Do specific peculiarities of human psychology play a role in ideological dynamics?

**Fig 1:**
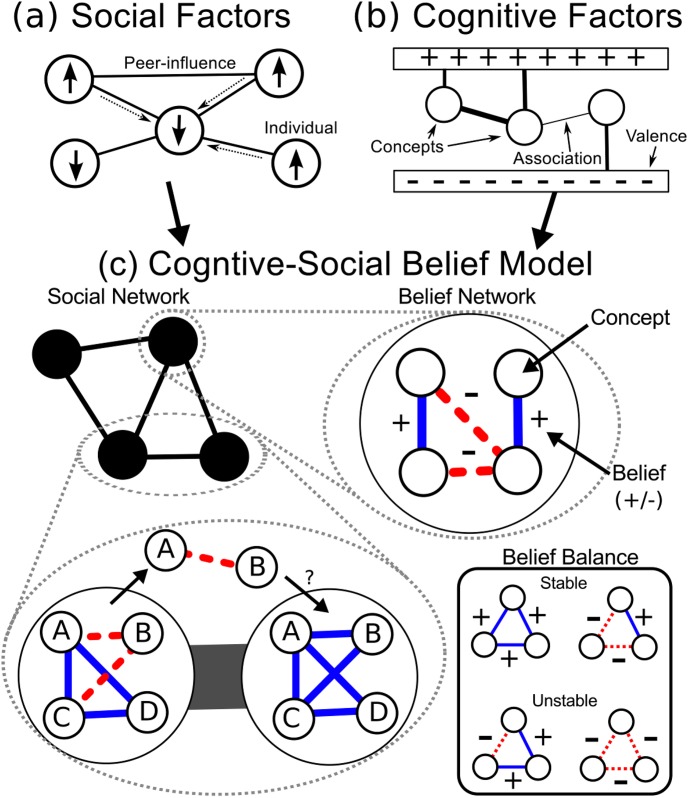
Cognitive-Social Belief Model. (a) Social models, such as the voter or Sznajd models, focus on the assimilation process through social pressure. Beliefs are usually simplified as independent states. (b) Cognitive models, such as the SKS model, focus on the interaction and coherence of beliefs of a single individual and how individuals make decisions and change their minds. The effect of social networks is often unaddressed. (c) Our model incorporates both forces, recognizing not only social pressures but also the connected nature of human beliefs. The social network acts as a conduit for belief transmission between individuals. We model a belief as a signed relationship between two concepts. We express the internal coherence of a network of such beliefs in terms of social balance theory where relationship triads can be either stable or unstable. The belief networks evolve over time as individuals decide whether to accept new beliefs transmitted by their peers.

Although these questions have been tackled by numerous studies, most existing models of belief system dynamics focus on either social or cognitive factors rather than integrating both aspects. Social models focus on how social interactions transmit and shape beliefs. For instance, Axelrod’s cultural dissemination model considers social influence and homophily as key drivers of cultural polarization (see Fig 1A) [6, 7]. In this model each agent is represented as a vector of independent traits that can be modified through social influence. The study of spin systems in physics have inspired a number of opinion models, such as the voter model [8–10], Sznajd model [11–13], and Ising-like models [14]. Other approaches have drawn upon reaction-diffusion systems [15], or may use continuous opinions [16] or bounded-confidence [17].

Cognitive modeling approaches mainly focus on information processing and decision making (see Fig 1B) [18]. Psychological research has revealed that individuals strive for internal consistency, which leads to cognitive mechanisms such as *confirmation bias* [19] and *cognitive dissonance* [20]. Our understanding of these cognitive forces and biases in belief formation allow us to move beyond the underlying assumptions—a set of independent beliefs [21, 22]—of spin models or opinion vector-based models [23, 24].

Recent attempts to combine these forces have used mechanisms like social- and anti-conformity [13], foundational beliefs [25], confirmation bias [26], attitudes [27], and social network formation [28–30]. However, modeling belief-systems that consist of interacting beliefs, and the study of such systems under social influence, has not been fully investigated. Here we introduce a novel framework that can incorporate both social and cognitive factors in a coherent way (see Fig 1C). We show that the integration of social and cognitive factors produces elementary features of collective social phenomena—societal transitions, upheavals, and existence of fringe groups.

Our framework represents a society as a network of individuals—a social network—where each individual possesses a network of concepts and beliefs. Social influence takes place via the social ties [8, 31–33] and each individual carries a belief network of interconnected relationships that represents the individual’s belief system [20, 24, 34–38]. We evaluate the internal coherence of each individual’s belief network by applying the balance theory [39, 40]. The coherence shifts an individual’s willingness to integrate new beliefs, thus simulating cognitive traits such as confirmation bias and cognitive dissonance [18, 19, 23, 31–33]. At the same time, an individual’s belief network is affected by social influence; repeated exposure to new ideas through social ties [41–44] increases their likelihood of adopting even incongruent ideas. Individuals thus experience both cognitive and social forces: (i) they prefer to have coherent belief networks and prefer beliefs that will increase their internal coherence; but (ii) may accept conflicting beliefs under the influence of strong social pressure.

## Methods

Our approach considers a network of concepts and beliefs where the nodes represent concepts and signed edges between them represents binary associative beliefs that capture the relation between two concepts (cf. Social Knowledge Structure (SKS) model [34]). This formulation allows us to define internal coherence through the principle of triad stability in social balance theory [45–48].

For instance, consider the beliefs of Alice, who is a devoted spectator of *soccer*. The *Eagles* are her favorite soccer team, but it has been charged with *match fixing*. In Alice’s belief network there is a positive link between the *Eagles* and *soccer*, and between the *Eagles* and *match fixing*, while there is a negative link between *soccer* and *match fixing*. Such pressured configurations are considered unstable (incoherent), and are analogous to frustrated states in spin-systems or unstable social triads. To resolve the frustration Alice may dissociate the *Eagles* from the allegations of match fixing, drop the *Eagles*’ association with the sport, or change the relationship between *soccer* and *match fixing*. It has been shown that people tend to quickly resolve such inconsistency when provided the opportunity to choose dissonance reduction strategies [49]. Yet, in the presence of social pressure or more complicated concept associations, a concept may remain pressured. Each triad in a belief network can be either stable or unstable, as shown in Fig 1C. The incoherence of an entire belief system (of individual *n*) can thus be captured by an internal energy function [50] on the belief network M:

En(i)=-1M3∑j,k,lajkaklajl, (1)

where *M* is the number of nodes in the belief network and *a*_*jk*_ is the association connecting nodes *j* and *k*, which can be +1 (positive association) or −1 (negative association). The sum is taken over all triads in the belief network and normalized by the total number of triads. For simplicity, in our simulations we choose this network to be complete, meaning that all concepts have a positive or negative association with every other concept.

A single association’s contribution to the energy depends on the state of adjacent associations, providing interdependence and rigidity to the belief system. Beliefs do not necessarily reflect reality. They may be fabricated or completely false. It is, however, the *interaction* between beliefs that gives them their strength, reflective of psychological factors like confirmation bias and cognitive dissonance.

The evolution of belief systems is also driven by social interactions, through which people communicate their beliefs to others. We represent this society as a social network, N, where N=|N|, and whose nodes are individuals and edges represent social relationships through which ideas are communicated. We define a second, “social” energy term, inspired by energy in the spin-based models which captures the degree of alignment between connected individuals. The ‘local’ social energy that an individual n∈N feels can be defined by:

En(s)=-1kmaxM2∑q∈Γ(n)S→n·S→q, (2)

where the sum is taken over the set of *n*’s neighbors in N, denoted by Γ(*n*). $\vec{S}$ is a belief state vector where each element corresponds to an edge in the belief network, so |S→|=(M2). *k*_max_ is a normalization constant that bounds the strength of peer-influence and is equal to the maximum degree of N. Alternatively, it could be replaced by a function that specifies the scaling relationship between exposure and the individual’s energy. For our simulations everyone possesses the same set of concepts (nodes) so *M* is the same for each person.

We combine the internal energy with the social energy for all individuals to define the total energy as follows:

$$H=\sum_{n\in N}\left[JE_{n}^{\left(i\right)}+IE_{n}^{\left(s\right)}\right]$$ (3)

where the last sum is taken over all nodes on the network. The parameters *J* and *I*, which we refer to as the *coherentism* and *peer-influence* respectively, control the relative contribution of the internal energy and the social energy to the total. The dynamics is dominated by internal belief coherence if *J* ≫ *I* and by social consensus when *I* ≫ *J*.

Each individual is endowed with their own internal belief network and may transfer some of their beliefs to their social contacts. A receiver of a belief either accepts the incoming belief or not based on the context of their own belief system (internal coherency) and similarity to their neighbors (social conformity). A belief is more likely to be accepted if it increases the coherence of an individual’s own belief system, social pressure will also increase the odds of a belief being accepted, even if it conflicts with their belief system.

We implement these ideas by creating the following rules: at each time step *t*, a random pair of connected individuals is chosen and one of the individuals (sender) randomly chooses a belief (association) from its internal belief system and sends it to the other individual (receiver), as illustrated in Fig 1C. We assume that each individual has an identical set of concept nodes. Fig 1C shows the selection and emission process on a graph. The receiver accepts the association if it decreases their individual energy: $H_{n}=JE_{n}^{\left(i\right)}+IE_{n}^{\left(s\right)}$. Even if the change in energy is less than zero, Δ*H*_*n*_ > 0, the receiver may still accept with the probability of $e^{\frac{-\Delta H_{n}}{T}}$. This term is analogous to the Boltzmann factor [51]. *T*, which we refer to as *susceptibility*, serves a similar purpose as temperature in physical systems for the belief network. As *T* increases, an individual is more likely to accept their neighbor’s opinions that conflict with their own.

We characterize the status of the whole society by defining two global energy functions. First, the *mean individual energy* 〈*E*^(*i*)^〉 measures the average internal coherence of individuals. It is expressed by the following equation:

$$\begin{array}{c} \left\langle E^{\left(i\right)}\right\rangle =\frac{1}{N}\sum_{n\in N}E_{n}^{\left(i\right)}. \end{array}$$ (4)

The average is taken over the energies of all individuals and it can take values between +1 and −1. 〈*E*^(*i*)^〉 = −1 means that every individual in the society possesses a completely coherent belief system, with no pressured beliefs. The other extreme (+1) represents a society where every individual has completely incoherent beliefs.

Yet, this measure does not give us any indication of how homogeneous a society is, as belief systems can vary widely while still being coherent. We have a second energy measure inspired from spin systems:

$$\begin{array}{c} \left\langle E^{\left(s\right)}\right\rangle =\frac{1}{N}\sum_{n\in N}E_{n}^{\left(s\right)}, \end{array}$$ (5)

which is minimized if the society is in consensus.

For each simulation we use Erdös-Rènyi graphs with *N* = 10^4^ nodes and average degree of 5, though similar results are found for 2D lattices. The belief network was fully connected with *M* = 5.

## Results

Most opinion models exhibit a phase transition from a disordered state to an ordered one [8], where the ordered state represents consensus. As our model includes the two conflicting forces—personal belief rigidity and social influence—we first ask how the relative strength of these two forces governs consensus dynamics.

**Fig 2:**
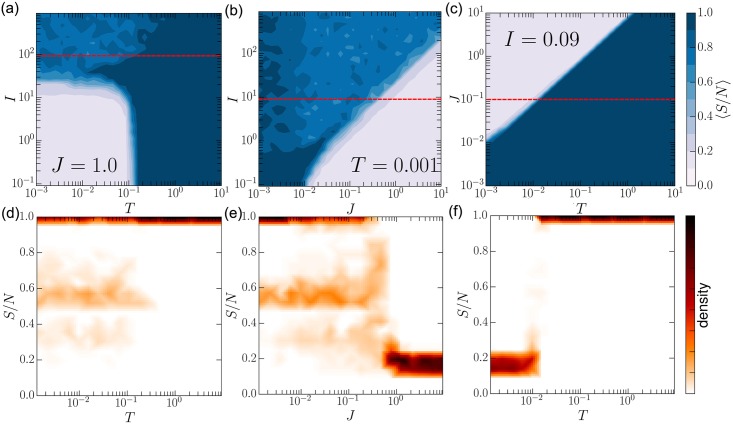
Phase space. (a-c) Phase diagrams of various combinations of the three parameters *J*, *I*, and *T*. Along with corresponding slices through the phase space as indicated by the dashed red lines (bottom row). (a,d) peer-influence *I* and susceptibility *T* conflict creating a regime where multiple belief systems with various coherences can coexist. We see a similar regime appear in (b, e) where peer-influence and coherentism contend for dominance. More traditional disorder-to-order transitions as in other opinion models also take place when *I* is small and fixed (c, f). *S*/*N* is the fractional size of the largest group. ER graphs with *N* = 10^4^ nodes and average degree of 5 were used. The density was calculated from a 160 trails per point. The belief network was fully connected with *M* = 5.

Through social interaction, the society may or may not reach a consensus, depending upon the relative strength of peer-influence (*I*) and coherentism (*J*). Fig 2 shows a set of phase diagrams for various combinations of *J*, *I*, and *T*. *S*/*N* is defined as the belief system with the largest number of constituents normalized by the size of the social network. Density in Fig 2D–2F is the probability density of *S*/*N* over 160 trials at a given parameter configuration. Since belief systems evolve locally, individuals may have to pass through incoherent states before fully converting. As the coherentism (*J*) increases individuals tend to cling to their own beliefs rather than make such incoherent transitions. This can prevent consensus, but small local consensus can still occur. As the susceptibility (*T*) increases individuals readily accept incoherent beliefs, allowing individuals to traverse through the belief space. It facilitates spreading of ideas and consensus. By making individuals more susceptible to belief spreading rather than to random switching, *T* plays the opposite role to temperature in standard spin models [8]. *T* acts as a temperature for individual’s beliefs, and as an inverse-temperature for the whole system. As the peer-influence (*I*) increases individuals again become more prone to consensus. Novel dynamics occur when the effect of coherentism and social influence become comparable. Fig 2B shows that complete consensus is not guaranteed when the forces exerted by *J* and *I* remain comparable in size. The competition leads to a situation where belief systems can coexist and where less coherent systems can dominate. In Fig 2D and 2E, we see examples of these competing configurations for when T or J is decreased and I held constant, as denoted by the higher density of largest belief-systems around 0.5. In these cases there are usually two (sometimes more) larger competing belief systems that cannot completely dominate the system even after a very long time (hundreds of billions of time-steps). As noted in [50] there are local minima distributed throughout belief space where an individual’s belief system can get stuck. At lower temperatures and when the drive for internal coherence (J) is much smaller than I, then groups of individuals (sometimes the whole population) can collectively become stuck in these “jammed” states. Under these conditions convergence to a completely coherent belief system is not guaranteed.

In our model the energy contribution of belief networks can have a major impact on system stability as individuals seek more coherent beliefs and resolve dissonance. What happens when key beliefs are upset through an external shock or perturbation?

**Fig 3:**
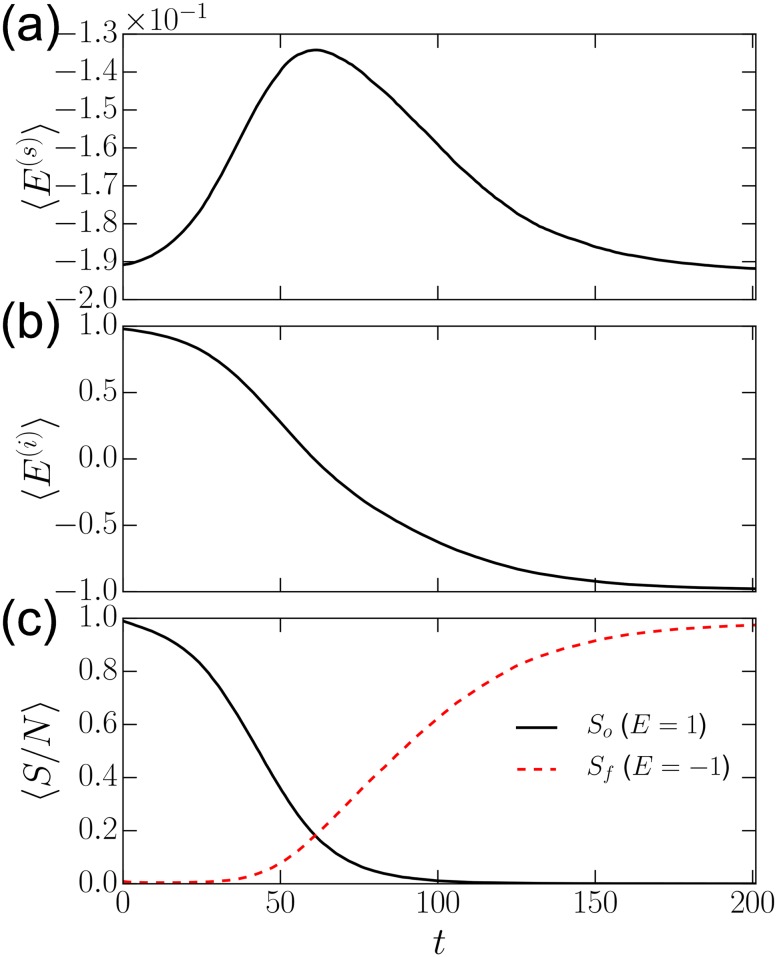
Belief driven social instability. Strong societal consensus does not guarantee a stable society in our model. If major paradigm shifts occur and make individual belief systems incoherent, then society may become unstable. (a) The plot shows the evolution of social energy *E*^(*s*)^ over time. The system starts at consensus but with incoherent beliefs. After introducing a small perturbation, individuals leave consensus, searching for more coherent sets of beliefs, until society re-converges at a stable configuration. (b) Decreasing mean individual energies 〈*E*^(*i*)^〉 over time illustrates individual stabilization during societal transition. (c) 〈*S*/*N*〉 is the fractional group size. As society is upset, the original dominant but incoherent belief system *S*_*o*_ (solid black) is replaced by an emerging coherent alternative *S*_*f*_ (dashed red).

Imagine two independent systems, both homogeneous with the same social energy, *E*^(*s*)^. In traditional social models, with the absence of the individual belief system (cognitive factor), these systems are identical. By contrast, the internal system of interconnected beliefs introduces a new force that drives people to seek coherence in the structure of their own belief systems. Given a homogeneous population of people with *highly coherent* belief systems, society remains stable. However, given a homogeneous population of *incoherent* belief systems, society will become unstable and following a small perturbation, breaks down (see Fig 3). In our simulation, the society is initialized at consensus with an incoherent belief system. Then 1% of the population are given a random belief system. Individuals attempt to reduce the energy of their own belief systems and leave consensus. This society eventually re-converges at a more coherent belief-system that is different from the original consensus (Fig 3C). In the model, consensus does not guarantee stability.

These social instabilities may help explain why new ideologies can arise in the presence of belief systems that dominate most of the population. Traumatic events such as war or depression could make previously coherent beliefs less coherent, thereby destabilizing the belief system as a whole. Since these beliefs are shared by the overwhelming majority, this perturbation has the potential to have widespread impact. Such changes may reduce the internal coherence of individuals, which may make the whole society more prone to paradigm shifts. Throughout history, major external perturbations in the form of war, depression, and crippling inflation has frequently disrupted the world-views of a society’s citizens, inducing subsequent social upheavals. More coherent belief systems, which otherwise fail to gain adherents in the presence of a dominant stable societal values, could then gain the upper-hand by recruiting among a population of disturbed citizens as Hoffer suggests [2].

Given that the coherence of personal beliefs fundamentally impacts collective behavior, we investigate the impact of “true believers” or zealots in a population. They can play an important role in shaping collective social dynamics [2, 8]. Zealots were introduced in [52] and [53] in the context of the voter model. Since, there has been continued work on the impact of zealots in the voter model [54–56] as well as binary adoption [57], naming game [58, 59], and other social models [60, 61]. The related topic of minority spreading has also been explored in various opinion models [62–64]. Here we explore this aspect of opinion dynamics from the perspective of cognitive forces acting on the agents. In the context of our framework we define zealots as individuals who will never alter their own belief systems, but will continue to attempt to convert others to their own.

**Fig 4:**
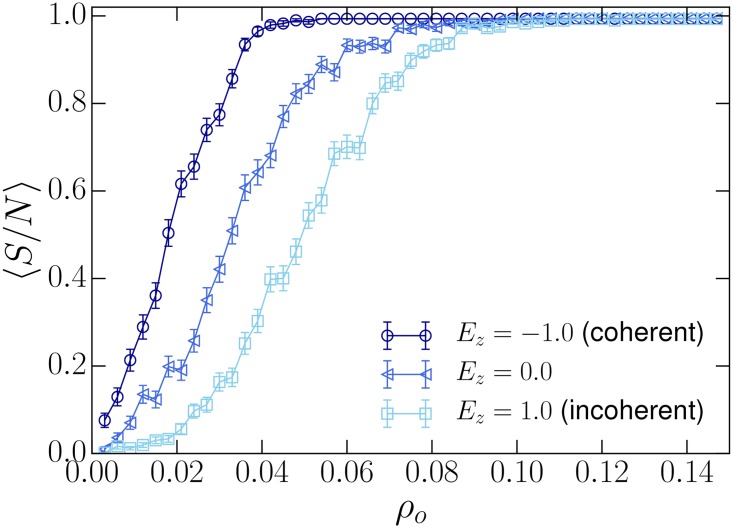
Impact of the internal consistency of zealot beliefs. We define zealots as a group of individuals who share an identical, immutable belief system. Such belief systems can, however, vary in terms of their coherency. The dynamics of 〈*S*/*N*〉, the fractional size of the zealot population, over *ρ*_*o*_, the density of zealots introduced into the population, reveals that zealots with more coherent beliefs can convert a population much more efficiently. In converting the whole population, the coherent set of beliefs (circles) require only less than half the density of zealots compared with incoherent beliefs (squares). Bars show standard error and *E*_*z*_ is the energy of the zealot’s belief-system. The simulations were run using *J* = 2.0, *T* = 2.0 and *I* = 90.0.

To study the impact of zealots, we prepare a homogeneous society with highly coherent beliefs and introduce zealots with varying internal coherence. As seen in studies of previous social models, there is a *tipping-point* density above which the minority opinion takes over the population. Fig 4 shows that the internal coherence of zealots has a strong impact on their effectiveness in converting society. Low coherence zealots require much higher densities in order to convert the whole population (see squares in Fig 4) because converted individuals revert back to more coherent belief-systems at a higher rate, making it difficult for the zealot’s belief-system to retain converts. Highly coherent zealots pull the whole population out of consensus and convert it to their belief-systems more easily. Coherent zealots require almost half the density, in this particular case, to convert the entire population. This suggests that coherence of a set of beliefs plays a vital role in determining how well the set of beliefs spread through society.

Our model may offer a possible explanation for the co-existence of many seemingly invalid or impractical cults and fringe groups in our society. The beliefs of cults and other fringe groups may frequently contradict reality, yet they continue to thrive in spite of being surrounded by large majorities of other belief systems. In our model, this is explained by the degree to which the coherence of belief systems can out-balance social pressure in addition to social isolation.

**Fig 5:**
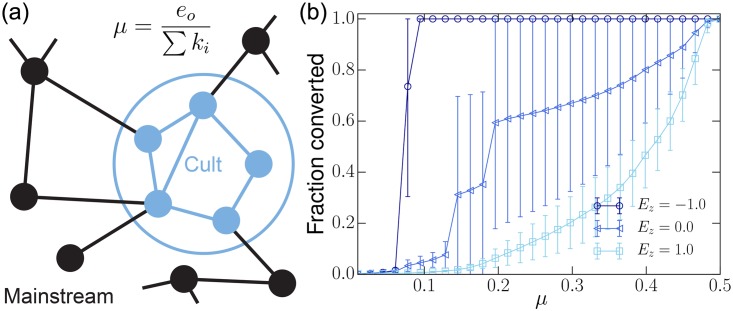
Belief Invasion. (a) The survival of cults and fringe groups depends on the coherence and strength of beliefs. We create a network with two communities with parameters *T* = 2.0, *I* = 0.09, and *J* = 2.0—putting the system in a regime where it will seek consensus. We vary the fraction of links that connect the cult community to the mainstream community, denoted *μ*. *e*_*o*_ is the number of social links between communities and ∑*k*_*i*_ is the total number of links in the cult (both shared and internal). The mainstream community attempts to convert the smaller cult. (b) At low *μ* the lack of exposure allows the cult to resist mainstream conversion. At higher *μ* there is sufficient exposure to the mainstream community to overcome the rigidity of the cult’s belief system. However, the process of conversion becomes more difficult as the cult’s beliefs become more coherent than mainstream beliefs. Cults are easily converted with highly coherent mainstream beliefs even at low exposure levels (black circles), while cults maintain their beliefs even at high exposure given low coherence of mainstream beliefs (red squares). Bars show standard deviation.

To investigate the influence of belief rigidity and social isolation on social dynamics we create a society with two communities, a large mainstream community and a smaller cult community (Fig 5A). The *mainstream* community acts like a reservoir of mainstream beliefs. The other community consists of people following a different coherent belief system. By controlling social exposure and the strength of belief coherence, we investigate what effect belief coherence has on the capacity for mainstream society to invade the cult. The parameter *μ* controls the fraction of edges in the cult community that are shared with the mainstream community [65]. When socially isolated (low *μ*) the cult can resist invasion regardless of mainstream coherence (Fig 5B). Groups of like-minded individuals can resist outside influence by reducing social contact with non-members (decreasing *μ*) and enhancing internal social interactions, both of which are common in many fringe groups and even in religious communities. On the other hand, the internal coherence also plays a key role. Less coherent mainstream beliefs have greater difficulty in converting the cult, even at high levels of exposure (high *μ*). Compared with the dogma of cults, the truth or the reality can be more complex and less coherent. In such case, even a belief-system firmly grounded on truth may struggle to convert cults that possess a highly coherent set of beliefs. Interlocked beliefs turns tightly-knit communities into bastions of resistance to ideological invasion from outside.

**Fig 6:**
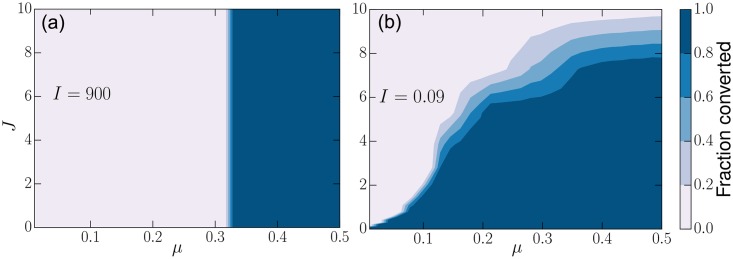
Community and belief rigidity. (a) Exposure determines conversion resistance when peer-influence (*I*) is strong. (b) Fringe groups can sustain their beliefs, even at a very high level of social exposure, with high levels of individual coherentism (*J*).

Fig 6A shows a phase diagram of the case when *I* ≫ *J*, where conversion is determined solely by social exposure (*μ*). This is expected by most spin-based social models. However, if the strength of social influence is comparable to belief strength, social exposure is not the sole determinant anymore (see Fig 6B). Notably, by increasing the coherentism (*J*) of its members and maintaining a coherent set of beliefs, cults can continue to thrive even with *complete mixing* with society. This contradicts, while underlining common intuition, the traditional exposure models where enough exposure is sufficient to convert populations. These results also hint at common characteristics of surviving cults. We do not expect to find successful cults that have low belief coherence and high mixing because they would be quickly converted by the mainstream society. We expect to see more cults that utilize a combination of policies that minimize their member’s social contacts with outsiders, emphasize the importance of their dogma (increasing *J*), and maximizing the coherence of their beliefs. The latter could mean incorporating explanations for beliefs that contradict empirical evidence or belittling mainstream methods of reasoning. Coherence can be, but is not necessarily aligned with logical consistency, rather coherence is based on the strength of associations between concepts and valence concepts (see SKS model [34]), that is derived from a connectionist cognitive framework.

## Discussion

We have shown that our model exhibits a disorder-to-order transition similar to other opinion models, while also exhibiting unique dynamics that could help explain common processes observed in the real world, such as the breakdown of a homogeneous society driven by shocks to individuals’ beliefs, the dependence of zealotry on belief coherence, and the successful entrenchment of fringe groups. Each of these results arises from the fact that our framework integrates belief interdependence with social influence.

The breakdown of homogeneous societies can manifest through changes in the connectivity of concepts at the concept-network level (cf. SKS model). Abrupt shocks to these conceptual connections can impact the coherence of beliefs. That impact is reflected in our model as an unstable belief system. When people share a common shock, one could expect that many of those people’s belief-systems will enter a frustrated state. In order for this socially frustrated state to be resolved, members of the population partake in societal-dissonance resolution strategies by collectively transitioning toward more coherent belief systems. From this view we could interpret paradigm shifts in culture as something akin to a societal-wide coping strategy.

Our model is a minimalistic approach toward introducing individualist concept-networks into a systematic social dynamics model. Though simple, the framework can be expanded to include more realistic features. For instance, communicated beliefs are chosen randomly, but this feature can be replaced with other models of belief communication, such as imitation, where someone will be more likely to communicate recently accepted beliefs. Additionally, our mapping from the SKS concept model could be expanded to included weighted relationships that emphasize the uncertainty that an individual has in their beliefs (conviction) and its relations to other beliefs. While we used the same parameter values (*J*, *I*, and *T*) for all individuals, we expect them to vary within the population from person to person. Variations of these parameters could produce the natural occurrence of zealot-like behavior. Finally, the formation and maintenance of social ties is an important facet of social dynamics [66, 67] and could be implemented by allowing agents to choose their neighbors [68].

Our model expands upon previous social models by implementing an internal belief system that assumes that the interdependence among beliefs shapes their dynamics. In this framework we are able to incorporate known psychological forces into the model and show that a bottom-up approach can successfully link micro-level behaviors to global dynamics. By making this small jump to using internal belief-systems, while preserving key features of standard social models, such as percolation and global consensus, it extends their dynamics and explanatory value, and could be a contributing factor for explaining the entrenchment of belief systems and social upheaval. Future work will be directed towards the development of new opinion models for specific applications that more closely integrate our behavioral and cognitive understanding of humanity.
